# Dataset on perception among male secondary school students on underage smoking in Jordan

**DOI:** 10.1016/j.dib.2020.105119

**Published:** 2020-01-13

**Authors:** Omar M.K. Mahasneh

**Affiliations:** Al-Balqa Applied University, Faculty of Shobak University College, Head of Department of Basic and Applied Science, PO box: (71911), Shobak (5), Maan, Jordan

**Keywords:** Curriculum and instruction, Secondary school students, Smoking, Underage, Jordan

## Abstract

The World Health Organization (WHO) revealed in 2015 that the percentage of smokers in Jordan is one of the highest in the world, reaching 70.2% among males and consequently ranking first in the Middle Eastern region and second in the world. Cigarettes are the most widely abused substance among school students in Jordan. This poses severe health risks to the public. The WHO emphasizes that one of the most important public health goals related to smoking is to reduce its harmful effects on the individual as well as society and the prevention and treatment of injuries. This article explores the nature of smoking among school students, binge smoking, and the consequences of smoking. Secondary school students undergo developmental transitions, and this comes with debilitating effects such as the risky use of cigarettes, which adversely affects their health and educational achievements. This article comprises data obtained from 1166 participants (ages 14–17 years) from selected schools in Jerash, near Amman, Jordan. For data collection, a youth questionnaire on underage smoking was utilized. The article presents information on the participants’ smoking demographic. Analyses of the data can provide insights into the reasons for the smoking habits of the youth, the negative effects of smoking on school students, strategies to reduce smoking consumption, level of consumption of daily smokers, health issues caused by smoking, the prevalence of smoking, the effect of smoker parents on stimulating their children, and common smoking areas. The data will be useful for institutions dealing with prevalent health problems in society (Smoking causes health problems that affect students' learning) as well as benefit future researchers.

**Table:** Specification Table

Subject	Curriculum and Instruction, Educational Psychology
Specific subject area	Psychology of Learning and Education, Counseling Psychology
Type of data	Tables and Figures
How data was acquired	Field survey techniques were adopted for data collection
Data format	Raw, analyzed, Descriptive and Inferential statistical data
Parameters for data collection	Frequency and percentage
Description of data collection	The questionnaire was distributed to respondents and analyzed using SPSS
Data source location	The Ministry of Education, Jerash Governorate Education Directorate, Jordan
Data accessibility	The data is included in this article

**Table:** 

**Value of the Data** • The details of the data can be used to strategize on how to reduce underage smoking in Jordan and the data can be compared with that from other countries. • The data provided can prove useful in analyzing the age differences within the demographic in relation to the volume of smoking. • The data can be used by counseling psychologists working with senior secondary school (high school) students. • The data may serve as a heuristic basis for future research on smoking. • The data can assist with planning for public health interventions.

## 1 Data description

**Fig. 1:**
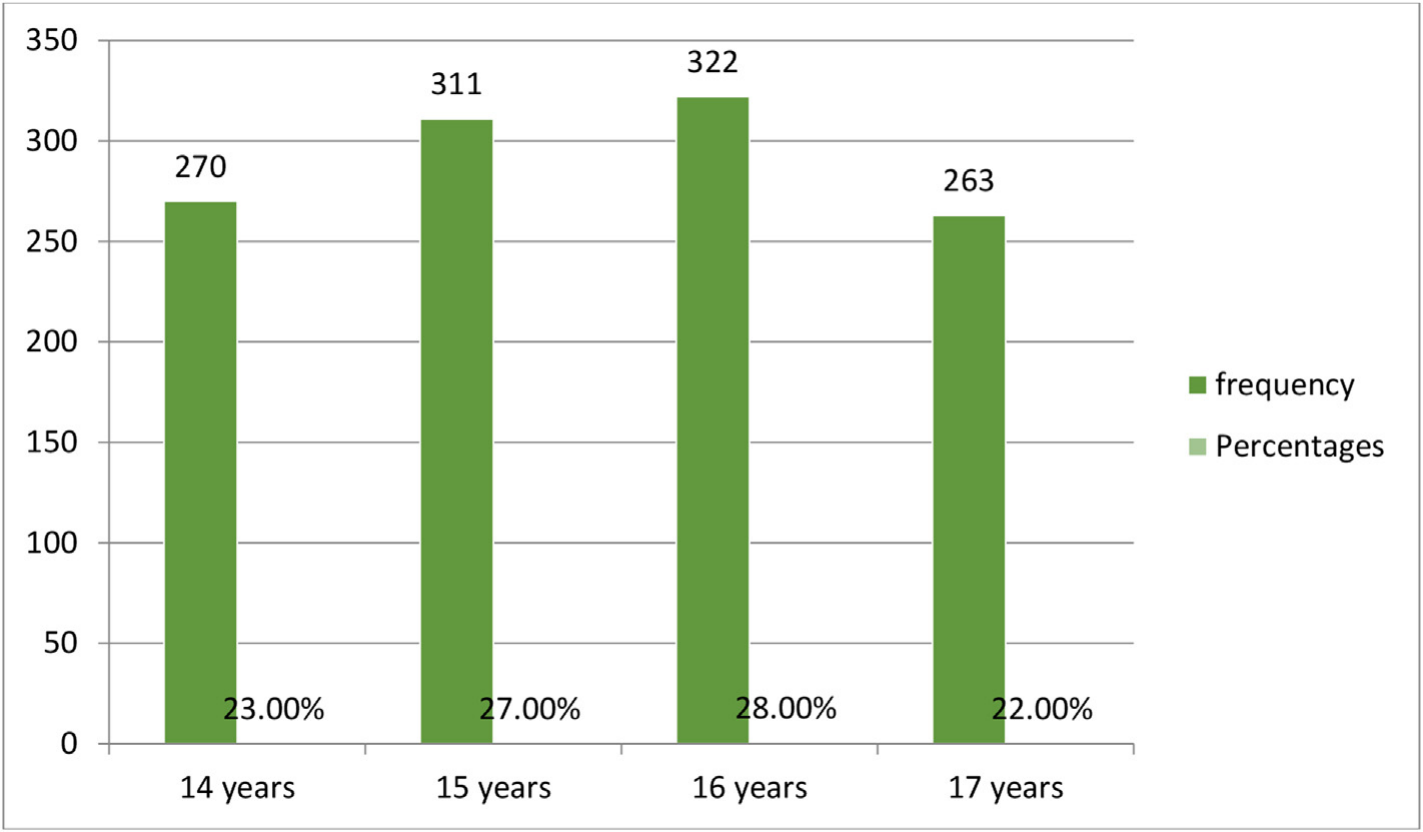
Distribution of respondents by age.

The data presented below was obtained through a structured questionnaire. The number of respondents involved in the survey was 1166. Fig. 1 shows the frequency of distribution by age: 270 (23%) – 14 years, 311 (27%) – 15 years, 322 (28%) – 16 years, and 263 (22%) – 17 years. Among the respondents, there were 914 (78.3%) smokers and 252 (21.7%) non-smokers (Table 1). Table 2 represents data on the frequency of cigarette consumption. Data derived in relation to the question ‘Does smoking cause health problems?’ shows that 876 respondents agreed and 38 disagreed (Table 8). Table 9 illustrates the data collected on the prevalence of smoking, based on the age of the smokers, to check whether it has increased, decreased, or remained at the same level. Table 3 illustrates data regarding the question ‘Do your parents smoke?’. The respondents also answered questions about the reasons for youth taking up the habit of smoking, and the most common answers were “Parental stimulation,” “To appear strong,” and “Family problems” (Table 4). The collected data revealed that the most common areas for smoking among school students were places around the school (Table 5), and the most common answer for the negative consequences of smoking was “Unpleasant odor” [6]. Table 7 shows that the most common strategy considered to reduce cigarette smoking was “Educate students in schools about the harmful effects of smoking.” The researcher relied on the following educational literature in writing the research [[1], [2], [3], [4], [5], [6], [7]].

**Table 1:** The frequency of smokers according to the respondents’ ages.

Variable		Do you smoke?		Total
		Yes	No	
Gender	Male	914	252	1166
Age	14 years	130	140	270
	15 years	211	100	311
	16 years	317	5	322
	17 years	256	7	263
Total		914	252	1166

**Table 2:** The frequency of cigarette consumption based on the respondents’ ages.

Variable		Frequency of smoking				Total
		1–5 cigarettes per day	6–10 cigarettes per day	11–20 cigarettes per day	Over 20 cigarettes a day	
Age	14 years	125	5	0	0	130
	15 years	200	10	1	0	211
	16 years	296	14	7	0	317
	17 years	200	15	40	1	256
Total		821	44	48	1	914

**Table 8:** The data collected for the question ‘Does smoking cause health problems?’ based on the respondents' ages.

Variable		Does smoking cause health problems?		Total
		YES	NO	
Age	14 years	125	5	130
	15 years	201	10	211
	16 years	305	12	317
	17 years	245	11	256
Total		876	38	914

**Table 9:** The prevalence of smoking based on the respondents’ ages.

Variable		Prevalence of smoking			Total
		Increased	Decreased	Stayed the same	
Age	14 years	70	20	40	130
	15 years	180	15	16	211
	16 years	256	50	11	317
	17 years	214	20	22	256
Total		720	105	89	914

**Table 3:** The data collected on the parents' smoking habits based on the respondents’ ages.

Variable		Do your parents smoke?				Total
		My father smokes		My mother smokes		
		Yes	No	Yes	No	
Age	14 years	120	10	4	126	130
	15 years	198	13	3	208	211
	16 years	302	15	6	311	317
	17 years	236	20	1	255	256
Total		856	58	14	900	914

**Table 4:** The data regarding reasons for cigarette smoking among youths.

Reason	Frequency/(%)	Rank
To appear strong	860 (27%)	1st
Parental stimulation	700 (22%)	2nd
Family problems	600 (18%)	3rd
Increased self-confidence	550 (17%)	4th
Peer pressure	500 (16%)	5th

**Table 5:** The data regarding preferred smoking places.

Preferred places	Frequency/(%)	Rank
Around the school	900 (30%)	1st
In the market	850 (29%)	2nd
In parks and cafes	740 (25%)	3rd
In the garden of the house	400 (14%)	4th
At home	70 (2%)	5th

**Table 6:** The data collected on the negative consequences of cigarette consumption.

Negative Consequences	Frequency/(%)	Rank
Unpleasant odors	910 (18%)	1st
Family problems	820 (17%)	2nd
Constant headaches when not smoking	800 (16%)	3rd
Poor concentration in school	720 (14%)	4th
Tooth decay	610 (12%)	5th
Sustained health problems	600 (12%)	6th
Severe cough	540 (11%)	7th

**Table 7:** The data collected on different strategies to reduce smoking consumption.

Approaches to decrease cigarette use	Frequency/(%)	Rank
Educate students in schools about the harmful effects of smoking	600 (30%)	1st
Activate the role of the media in raising awareness on the harmful effects of smoking	485 (24%)	2nd
Raising parents' awareness of the causes of smoking	479 (24%)	3rd
Prohibition of smoking in public places	436 (22%)	4th

## 2 Experimental design, materials, and methods

### 2.1 Research design

The research adopted a descriptive survey design to evaluate the dataset on the perception among male secondary school students on underage smoking in Jordan. This dataset included 1166 students from selected secondary schools in Jerash, Jordan. Fig. 1 Distribution of respondents by age.

### 2.2 Instruments

The researcher adopted the use of a questionnaire to collect data for this survey. This questionnaire had 11 questions including 7 specific questions and 4 open-ended questions based on the responses to the specific questions. The first question dealt with respondents’ socio-demographic characteristics (Appendix).

### 2.3 Instrument validity and reliability

The questionnaire was checked by experts for proper language, clarity, relevance, and comprehensiveness of the content, and a pilot survey was conducted to ensure that the questionnaire yielded consistent finding. This included a pretesting survey among male secondary school students in Jordan which is not included in the research sample to ensure accuracy in data.

### 2.4 Data sample

The data sample consisted of 1166, were chosen at random from all secondary schools.

### 2.5 Data analyses

The researcher coded the questionnaire questions and their answers, by number, according to the number of options available for each question. This was entered into an Excel sheet, and the data was analyzed using SPSS for frequency and percentage.

For instance, the first question was coded Q1, and the answers were coded as follows:

14 years was coded (1), 15 years was coded (2), 16 years was coded (3), and 17 years was coded (4).

A similar process was followed for the rest of the questions and their corresponding data.

### 2.6 Research questions

The data attempted to answer the following questions:

1. Why prompts underage male secondary school students in Jordan to smoke?
2. What are male secondary school students' most preferred places to smoke?
3. What are the negative effects of smoking on underage male secondary school students?
4. What are the optimal strategies to reduce cigarette consumption in male secondary school students?
5. Does smoking cause health problems in male secondary school students?
6. Is the number of cigarettes consumed per day by male secondary school students increasing, decreasing, or the same?

### 2.7 Dataset

The survey data was collected as presented in the following tables.

According to Table 1 the number of smokers (914) and non-smokers (252) from 1166 respondents, Distributed for ages (14–17 years). The researcher emphasizes the effect of smoking on the academic achievement of students as confirmed by a research [1,2].

According to Table 2 the number of smokers 1–5 cigarettes per day (125) 14 years, (200) 15 years, (296) 16 years and (200) 17 years. The researcher emphasizes that smoking causes health problems that negatively affect the achievement and learning of students in schools as confirmed by a researcher [[3], [4], [5]].

According to Table 3 the number of parents who smoke (130) 14 years, (211) 15 years, (317) 16 years and (256) 17 years.

### 2.8 The data questions

**What prompts underage male secondary school students in Jordan to smoke?**

According to Table 4 (27 %) of the respondents’ smoke “To appear strong”, (22%) “Parental stimulation”. (18%) family problems, (17%) “Increased self-confidence”, and (16%) Peer pressure.

**What are your most preferred places to smoke?**

According to Table 5 (30 %) of the respondents’ preferred smoking places “Around the school”, (29%) “In the market”. (25%) In parks and cafes, (14%) “In the garden of the house”, and (2%) At home.

**What are the negative effects of smoking on underage male secondary school students?**

According to Table 6 (18 %) of the respondents‘ negative consequences of cigarette consumption “Unpleasant odors”, (17%) “Family problems”. (16%) “Constant headaches when not smoking”, (14%) “Poor concentration in school”, (12%) “Tooth decay”, (12%) “Sustained health problems” and (11%) “Severe cough”. The researcher emphasizes the effect of smoking on the students ’focus in the classroom.

**What are the optimal strategies to reduce cigarette smoking in male secondary school students?**

According to Table 7 (30 %) of the respondents’ strategies to reduce smoking consumption “Educate students in schools about the harmful effects of smoking”, (24%) “Activate the role of the media in raising awareness on the harmful effects of smoking”. (24%) “Raising parents’ awareness of the causes of smoking and (22%) “Prohibition of smoking in public places”.

**Does smoking cause health problems in male secondary school students?**

According to Table 8 the number of smoking cause health problems sure (125) 14 years, (201) 15 years, (305) 16 years and (245) 17 years.

**Is the number of cigarettes consumed per day by male secondary school students increasing, decreasing, or the same?**

According to Table 9 the number of cigarettes consumed per day increased (70) 14 years, (180) 15 years, (256) 16 years and (214) 17 years. The researcher emphasizes the effect of smoking on the student's ability to retrieve information during the lectures as confirmed by a researcher [7].

## Funding

This data did not receive any external funding and was independently financed.

## Figures and Tables

**Table:** 

N	Dear Student, For scientific research, please answer the following questions.				
1.	How old are you?	14 years	15 years	16 years	17 years
2.	Do you smoke? If yes, answer the questions that follow:	YES		NO	
3.	Does your father smoke?	YES		NO	
4.	Does your mother smoke?	YES		NO	
5.	Does smoking cause health problems?	YES		NO	
6.	How many cigarettes do you consume daily?	1–5 cigarettes per day	6–10 cigarettes per day	11–20 cigarettes per day	Over 20 cigarettes a day
7.	What is the prevalence of cigarette consumption?	Increased	Decreased	The same	
8.	Why do you smoke? (Tick all that apply) Peer pressure Increased self-confidence Family problems Parental simulation To appear strong Other (please mention reason)				
9.	Where do you smoke**?** (Tick all that apply) At home In the garden of the house In parks and cafes In the market Around the school Other (please mention)				
10.	What are the negative effects of smoking on you? (Tick all that apply) Increased cough Sustained health problems Tooth decay Poor concentration in school Constant headache when not smoking Family problems Unpleasant odors Other (please mention)				
11.	In your opinion, what are the possible strategies for reducing smoking consumption? (Tick all that apply) Prohibition of smoking in public places Raising parents' awareness on the causes of smoking Activating the role of the media in raising awareness on the harmful effects of smoking on individuals Educating students in schools about the harmful effects of smoking Other (please mention)				
