# Supplementary material for: Dataset on perception among male secondary school students on underage smoking in Jordan
Source: Data Brief. 2020 Jan 13;29:105119. doi: 10.1016/j.dib.2020.105119 (PMC6997803; doi:10.1016/j.dib.2020.105119)
Supplement: Multimedia component 2 [file mmc2.docx]

**QUESTIONNAIRE**

| **N** | **Dear Student,**  For scientific research, please answer the following questions. | | | | |
| --- | --- | --- | --- | --- | --- |
| 1. | How old are you? | 14 years | 15 years | 16 years | 1. years |
| 2. | Do you smoke?  If yes, answer the questions that follow: | YES | | NO | |
| 3. | Does your father smoke? | YES | | NO | |
| 4. | Does your mother smoke? | YES | | NO | |
| 5. | Does smoking cause health problems? | YES | | NO | |
| 6. | How many cigarettes do you consume daily? | 1–5 cigarettes per day | 6–10 cigarettes per day | 11–20 cigarettes per day | Over 20 cigarettes a day |
| 7. | What is the prevalence of cigarette consumption? | Increased | Decreased | The same | |
| 8. | Why do you smoke? (Tick all that apply)  -----------Peer pressure  -----------Increased self-confidence  -----------Family problems  -----------Parental simulation  -----------To appear strong  ----------Other (please mention reason) | | | | |
| 9. | Where do you smoke**?** (Tick all that apply)  ----------At home  ----------In the garden of the house  ----------In parks and cafes  ----------In the market  ----------Around the school  ----------Other (please mention) | | | | |
| 10. | What are the negative effects of smoking on you? (Tick all that apply)  --------- Increased cough  --------- Sustained health problems  --------- Tooth decay  --------- Poor concentration in school  --------- Constant headache when not smoking  --------- Family problems  --------- Unpleasant odors  --------- Other (please mention) | | | | |
| 11. | In your opinion, what are the possible strategies for reducing smoking consumption? (Tick all that apply)  -----------Prohibition of smoking in public places  -----------Raising parents’ awareness on the causes of smoking  -----------Activating the role of the media in raising awareness on the harmful effects of smoking on individuals  ----------Educating students in schools about the harmful effects of smoking  ----------Other (please mention) | | | | |

**Thank you**
